# Distinct histone modifications denote early stress-induced drug tolerance in cancer

**DOI:** 10.18632/oncotarget.23654

**Published:** 2017-12-24

**Authors:** Abdullah Al Emran, Diego M. Marzese, Dinoop Ravindran Menon, Mitchell S. Stark, Joachim Torrano, Heinz Hammerlindl, Gao Zhang, Patricia Brafford, Matthew P. Salomon, Nellie Nelson, Sabrina Hammerlindl, Deepesh Gupta, Gordon B. Mills, Yiling Lu, Richard A. Sturm, Keith Flaherty, Dave S. B. Hoon, Brian Gabrielli, Meenhard Herlyn, Helmut Schaider

**Affiliations:** ^1^ Dermatology Research Centre, The University of Queensland Diamantina Institute, The University of Queensland, Translational Research Institute, Brisbane, Australia; ^2^ Department of Translational Molecular Medicine, John Wayne Cancer Institute, Santa Monica, CA, USA; ^3^ Sequencing Center, John Wayne Cancer Institute, Santa Monica, CA, USA; ^4^ The Wistar Institute, Philadelphia, PA, USA; ^5^ MD Anderson Centre, Houston, TX, USA; ^6^ Massachusetts General Hospital, Harvard Medical School, Boston, MA, USA; ^7^ Mater Research Institute, Translational Research Institute, The University of Queensland, Woolloongabba, Queensland, Australia

**Keywords:** acquired drug resistance, stress-induced resistance, histone modification, DNA methylation, epigenetic reprogramming

## Abstract

Besides somatic mutations or drug efflux, epigenetic reprogramming can lead to acquired drug resistance. We recently have identified early stress-induced multi-drug tolerant cancer cells termed induced drug-tolerant cells (IDTCs). Here, IDTCs were generated using different types of cancer cell lines; melanoma, lung, breast and colon cancer. A common loss of the H3K4me3 and H3K27me3 and gain of H3K9me3 mark was observed as a significant response to drug exposure or nutrient starvation in IDTCs. These epigenetic changes were reversible upon drug holidays. Microarray, qRT-PCR and protein expression data confirmed the up-regulation of histone methyltransferases (SETDB1 and SETDB2) which contribute to the accumulation of H3K9me3 concomitantly in the different cancer types. Genome-wide studies suggest that transcriptional repression of genes is due to concordant loss of H3K4me3 and regional increment of H3K9me3. Conversely, genome-wide CpG site-specific DNA methylation showed no common changes at the IDTC state. This suggests that distinct histone methylation patterns rather than DNA methylation are driving the transition from parental to IDTCs. In addition, silencing of SETDB1/2 reversed multi drug tolerance. Alterations of histone marks in early multi-drug tolerance with an increment in H3K9me3 and loss of H3K4me3/H3K27me3 is neither exclusive for any particular stress response nor cancer type specific but rather a generic response.

## INTRODUCTION

Tumours that are initially sensitive to a particular drug often develop resistance through a range of different mechanisms including somatic mutations, drug efflux, increased expression of the therapeutic target, DNA damage repair, cell death inhibition, activation of alternative signalling pathways, or epithelial-mesenchymal transition (EMT) [1-3]. Recent evidence indicates that drug resistance is often established without stable resistance-conferring genetic alterations [4, 5]. This entails alternative mechanisms such as epigenetic reprogramming for developing acquired resistance [6, 7]. Several studies have shown that cancer patients, who initially respond well to chemo- or molecularly targeted therapy but acquire resistance, can become re-sensitized to these same drugs by providing a “drug holiday”, a period without drug treatment [8, 9]. This is called ‘retreatment response’ [10, 11] and suggests that in some cases acquired resistance is a reversible effect. Moreover, we have previously found that transient but still reversible acquired drug resistance develops as a generic stress response rather than selection for a particular subpopulation demonstrating phenotypic plasticity contributing to drug resistance [12]. The underlying mechanisms and their relative contribution to cancers are still elusive. Initial studies on epigenetic modifications in cancer relied heavily on global DNA hypo-methylation [13] and later hyper-methylation at the promoter CpG island region of tumour suppressor genes [14]. Later on, histone modifications have been identified as one of the key epigenetic modifications in cancer [15]. Epigenetic modifications are not independent events rather they act in conjunction with chromatin modifiers like histone deacetylase (HDACs) and histone methyl-transferase (HMTs) maintaining particular histone marks [16]. These key epigenomic regulators are frequently dysregulated in different cancer types [17].

Based on our recent findings in melanoma [12], histone modifications and DNA methylation were studied in early stress-induced drug-tolerant cancer cells (IDTCs) from different cancer types. Our data suggest that IDTCs undergo an analogous inherent transcriptional reprogramming defined by a significant genome-wide downregulation of gene expression. Independent of the global DNA methylation remodelling, this response was defined by a significantly increased level of H3K9me3, a repressive histone modification at gene-associated polycomb repressive domains (PRDs) and decreased level of H3K4me3, an activating histone modification at gene promoters and H3K27me3, a transcriptional enhancer-repressive modification, irrespective of the treatment regime and cellular origin.

## RESULTS

### Transition from parental to induced drug-tolerant cells is a generic feature of cancer cells

**Figure 1:**
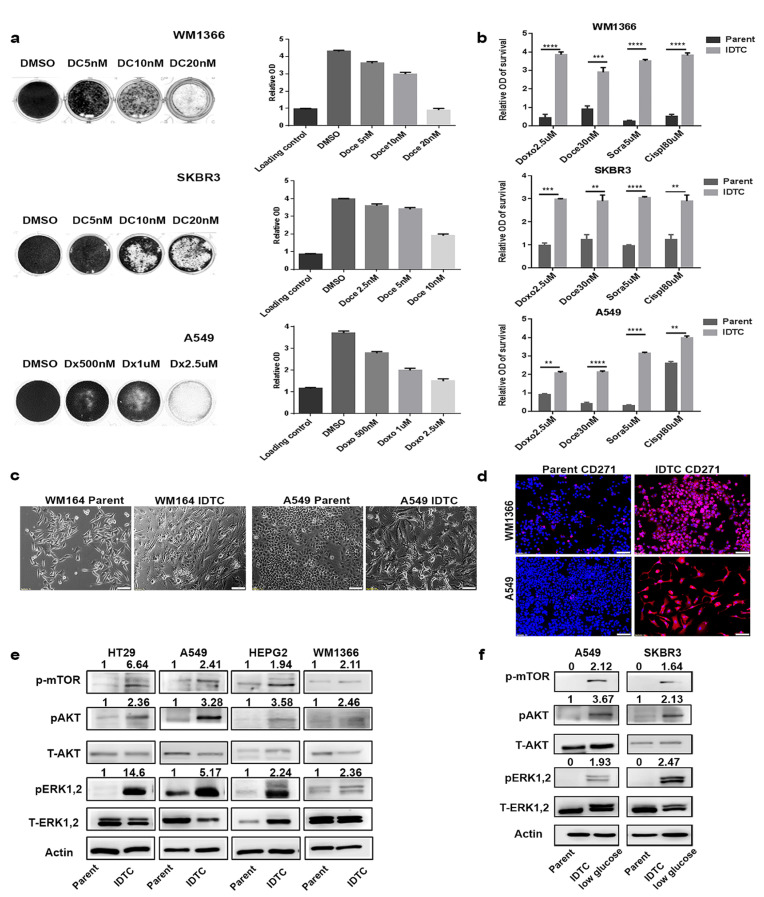
A common stress-induced transition of cancer cells into induced drug-tolerant cells (IDTCs) **a.** WM1366, SKBR3 or A549 cells were exposed to either dimethyl sulphoxide (DMSO), docetaxel (DC; 5nM, 10nM, 20nM; WM1366, SKBR3) or doxorubicin (Dx; 500nM, 1µM and 2.5µM; A549) for a period of 12 to15 days. Experiments were performed in biological triplicate. Surviving cells were stained with crystal violet (well images), then dissolved in 10% acetic acid and measured at 570nm to provide the relative cell number of viable cells. Crystal violet staining at day one after seeding was used as loading control compared with 12-15 days’ time point. Error bars represent the standard deviation from the mean. **b.** In a separate experiment, 5nM docetaxel and 500nM doxorubicin IDTCs were further exposed to higher concentrations of doxorubicin at 2.5µM, docetaxel at 30nM, cisplatin at 80µM and sorafenib at 5µM in biological triplicate. Relative survival of IDTCs compared to parental cells was measured by crystal violet staining after 72hrs. Statistical analysis was performed by unpaired t-test and P-value is represented as (*) where, ****P < 0.0001, ***P < 0.001, **P < 0.01, and *P < 0.05. **c.** Morphological changes during the transition of parental cells to IDTCs in WM164 and A549 cells (10x magnification). **d.** Expression of CD271 in WM1366 and A549 IDTCs compared to untreated control by IF. Representative CD271 (red) and Hoechst staining (blue) overlay images are shown. IgG isotype control is shown in Supplementary Figure 1e. (10x Magnification and bar is 100µm long) **e.** Protein lysates from IDTCs and the untreated control were subjected to immunoblotting for expression levels of the indicated antibodies. **f.** Indicated cell lines were maintained in low glucose (1mg/ml) containing media for 12-15 days. Cell lysates from low glucose IDTCs and normal glucose controls were probed for the indicated proteins. All western blot images were quantified by ImageJ software. Values were normalized by subtracting from loading control.

We previously demonstrated that *BRAF* mutant (mt) melanoma cells exposed to sub-lethal concentrations of a drug, hypoxia or nutrient starvation for 12 to 15 days convert into multi-drug tolerant cells [12]. These are termed induced drug-tolerant cells (IDTCs). To determine whether this innate response is cancer type specific, we generated IDTCs from *NRAS* mt melanoma (WM1366), *KRAS* mt lung (A549), *PIK3CA* mt breast (SKBR3), and *KRAS* mt colon (HT29) cancer cell lines by exposing them to sub-lethal concentrations of either chemotherapy or specific targeted therapies. WM1366 and SKBR3 IDTCs were generated using 5nM of docetaxel, A549 IDTCs with500nM of doxorubicin, and HT29 IDTCs using 25nM dabrafenib and 10nM of trametinib (Supplementary Table 1). Cell growth was determined after 12 days of drug exposure. Parental cells survived at lower sub-lethal concentrations such as 5nM docetaxel (WM1366 and SKBR3), 500nM or 1μM of doxorubicin (A549), and a combination of 25nM dabrafenib and 10nM trametinib (HT29) (Figure 1a and Supplementary Figure 1a). To assess the multi-drug resistance potential, IDTCs generated at 500nM doxorubicin and 5nM docetaxel were then exposed to a substantially higher dosage of the same drugs (2.5µM doxorubicin and 30nM docetaxel) in addition to 5µM of sorafenib and 80nM of cisplatin. In agreement with our previous observations [12], IDTCs generated from A549, WM1366, and SKBR3 survived at the higher dosage compared to their respective parental cells, demonstrating the development of multi-drug tolerance promoted by continued exposure to low drug concentrations (Figure 1b). As we have observed previously in melanoma, all IDTC lines developed from the four different cancer cell lines showed altered morphology compared to their parental cells. IDTCs had a more flattened, elongated and translucent phenotype compared to parental cells (Figure 1c and Supplementary Figure 1b). We have also demonstrated that CD271 is a characteristic marker of IDTCs in a *BRAF* mt melanoma model [12]. Concordantly, we observed that IDTCs from all four cancer cell lines showed a significant increase in CD271 expression (Figure 1d and Supplementary Figure 1c-d). Additionally, all IDTCs had increased activation of MAPK, AKT, and mTOR pathways, as previously demonstrated for the *BRAF* mt melanoma IDTCs [12] (Figure 1e). Other cellular stressors, such as low glucose media, also produced IDTCs with comparable signalling pathway activation (Figure 1f). These results were further corroborated by reverse phase protein array (RPPA) analyses of WM1366, A549, and HT29 IDTCs and their respective parental cells. RPPA results showed increased activation of JAK-STAT, AKT, and MEK signalling across the different cancer types suggesting a survival advantage of IDTCs over parental cancer cells (Supplementary Figure 1e).

### A distinct histone methylation pattern defines IDTCs

**Figure 2:**
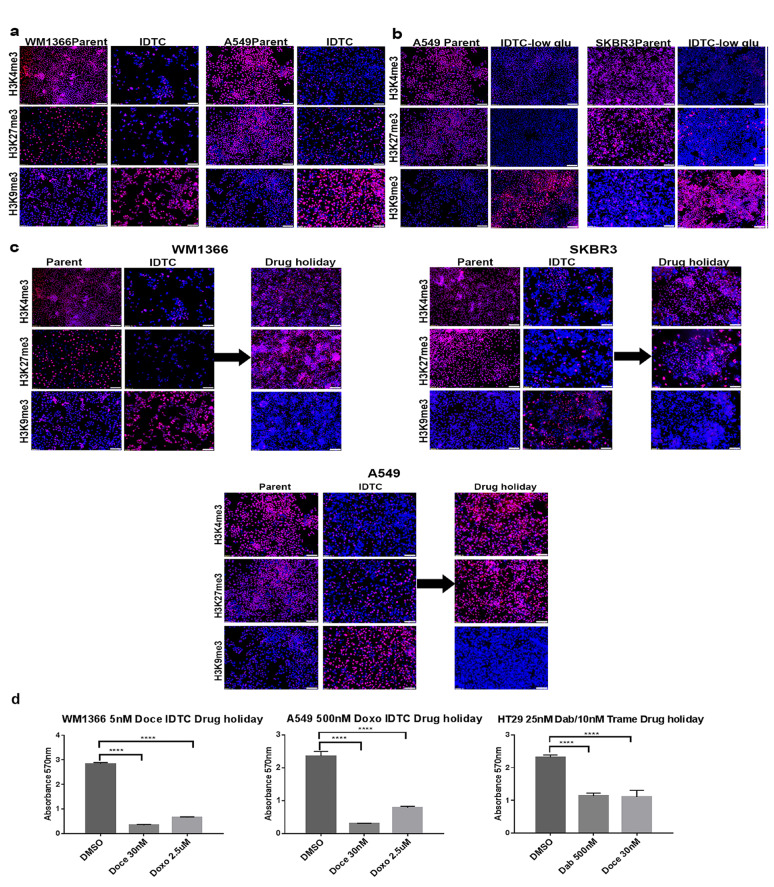
Stress and drug holidays dynamically modulate histone modifications **a.** WM1366, A549 IDTCs and their parental cells were tested for H3K4me3, H3K9me3 and H3K27me3 antibodies by IF. Representative antibodies staining and Hoechst (nuclear) staining were taken separately and overlaid picture are shown. **b.** A549 and SKBR3 cells were cultured with 1mg/ml glucose containing media for at least 12 days. Media was replenished after every three days. IF was performed for the indicated Abs to compare to parent cells supplemented with 5mg/ml glucose media. **c.** WM1366, SKBR3 and A549 IDTCs were allowed a drug holiday for ten days. During this period of time, no drug was provided and media changed every 72 hrs. Histone modifications were analysed by IF with the indicated Abs compared to corresponding parental cells. (All of the IF image were taken at 10x Magnification and bar is 100µm long) **d.** After 10 days of drug holidays WM1366, A549 and HT29 IDTCs were exposed to toxic concentrations of doxorubicin (2.5µM), docetaxel (30nM) for WM1366, A549 and dabrafenib (500nM) and docetaxel (30nM) for HT29. Cell survival was analysed by MTT assay. All experiments were done in triplicate. Statistical analysis was performed by the one-way ANOVA test and *P*-value is represented as (*) where, ****P < 0.0001.

We previously compared differences in gene expression between parental and *BRAF* mt melanoma IDTCs (WM164) and identified significant changes in epigenetic factors leading to an altered chromatin state [12]. Three histone demethylases involved in the removal of the active transcription mark H3K4me3 (KDM5A, KDM5B) and repressive mark H3K27me3 (KDM6A), as well as one histone methyltransferase (SETDB1) responsible for the establishment of the repressive H3K9me3 mark, were upregulated in IDTCs (Supplementary Figure 2a). To determine whether this is a common feature associated with the acquisition of drug tolerance, we evaluated these histone marks in IDTCs generated from different cancer types. We observed a consistent increase of H3K9me3 and decrease of H3K4me3 and H3K27me3 in IDTCs (Figure 2a and Supplementary Figure 2b-2c). Immunofluorescence (IF) quantification confirmed that H3K9me3, H3K4me3, and H3K27me3 showed significant differences (*P* < 0.0001) between parental cells and IDTCs (Supplementary Figure 2c). Stress due to nutrient starvation mimicked the histone modification patterns (*P* < 0.0001) suggestive of a generic stress response in cancer (Figure 2b). Additionally, two histone modifications H3S28ph and H4K16ac were tested which has been reported to be involved in acute cellular stress [18] and frequently lost in cancer respectively [19]. No significant differences were observed between IDTCs and parental cells (Supplementary Figure 2d and 2e).

### Drug holidays reverse histone alterations involved in the IDTC state

It has been demonstrated that a phenotypic switch as a result of drug holidays restores sensitivity to both chemotherapy and targeted therapy [11, 20]. We have demonstrated that drug sensitivity of IDTCs is restored by allowing drug holidays in melanoma cell IDTCs [12]. Importantly, drug holidays dynamically modulate histone modifications with an increase of the H3K4me3 and H3K27me3 mark and a decrease of the H3K9me3 mark akin the parental histone modification landscape in all cell types assessed (Figure 2c). This also corresponded to a phenotypic switch leading to re-sensitization to the drug they were originally desensitised to (Figure 2d).

### Common gene expression variations in IDTCs

**Figure 3:**
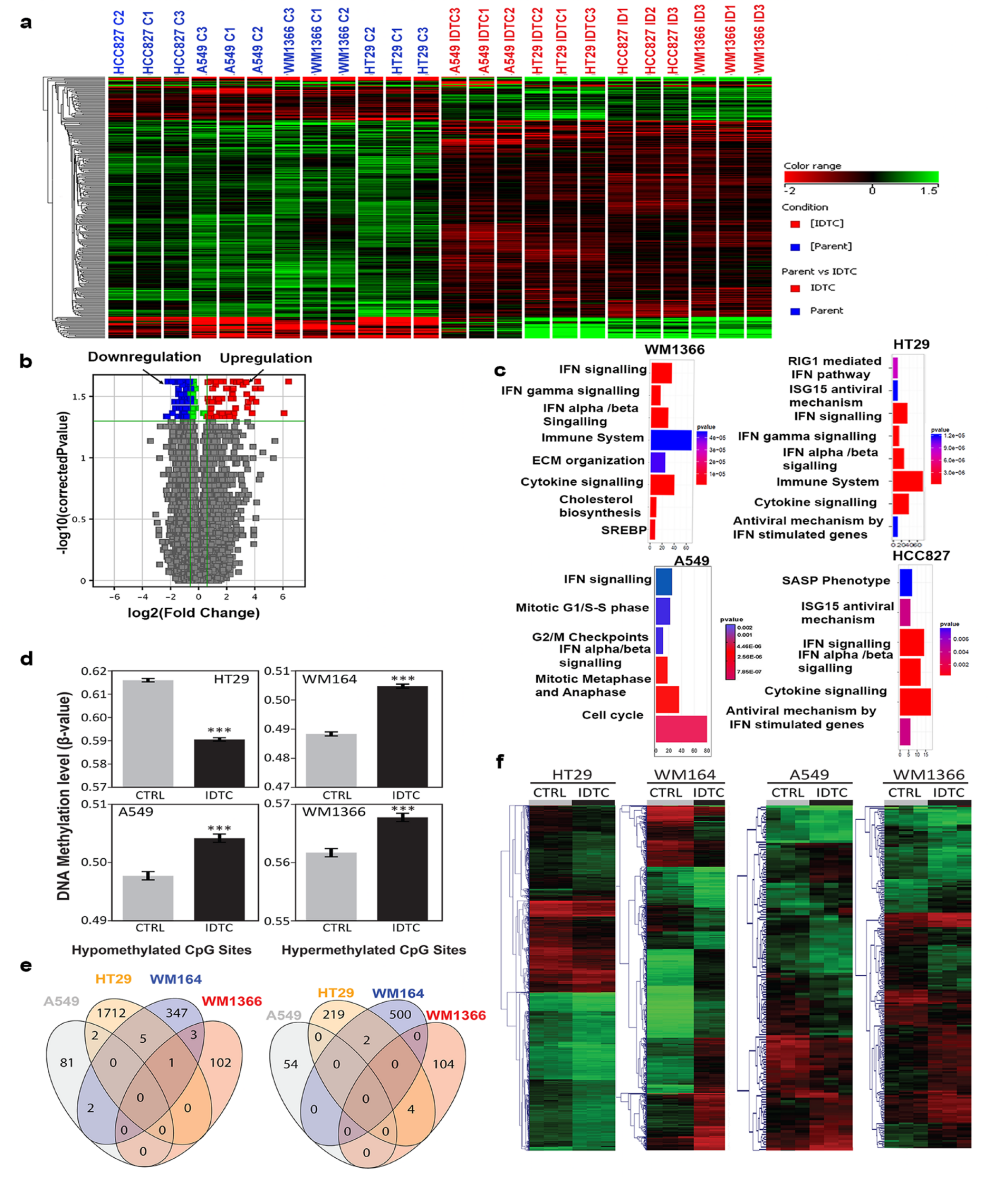
Differential expression of genes and genome wide DNA methylation in IDTCs compared to parental cells **a**. Hierarchical clustering of differential gene expression. Four cancer cell lines were analysed by the Australian Genome Research Facility (AGRF) for genome-wide differential expression using Illumina expression arrays. An unbiased hierarchical clustering was developed using the Mann-Whitney unpaired test with a fold change cut off FC≥ 1.5 and P≤0.05 as shown in the figure. **b.** A volcano plot was generated with a similar threshold as mentioned above depicting the overall 293 up and down-regulated genes. **c.** Pathways enriched for each of the IDTCs compared to parent cells are shown by using cluster Profiler7, with a p-value and a q-value cut off of 0.1 and 0.05 respectively. Genome-wide DNA methylation analyses reveal cell type-specific changes at the IDTC state. **d.** DNA methylation levels of IDTCs compared to untreated control. Bars represent the mean DNA methylation level for each cell line, with (IDTC) and without (CTRL) treatment. Error bars = S.E.M. **e.** Venn’s diagrams representing commonly differentially methylated (hypo- or hyper-methylated) CpG sites in IDTCs compared to control. **f.** Hierarchical clustering for cell-type using differentially methylated CpG sites. Differentially methylated CpG sites between the groups were identified using the Rank Products test [54]. After 100 permutations, CpG sites showing an adjusted *p*-value<0.05 (q-value) were considered significant. Scale bar for beta values: Green = 0 Black = 0.5 and Red = 1.

To identify differentially expressed genes in IDTCs, parental and IDTCs (WM1366, A549, HT29, and HCC827) were analysed for genome-wide expression by microarrays. Overall, there was a significant (*P* < 0.05) global down-regulation of gene expression in response to different drugs regardless of targeted or chemotherapy (Figure 3a, 3b and Supplementary Figure 3a). We identified cell type-specific and common gene expression variations affecting biological processes, cellular location, and molecular functions in IDTCs (Supplementary Figure 3b). Pathway enrichment analyses showed that genes involved in IFN-α, -β, -γ signalling, and cytokine signalling are altered in IDTCs (Figure 3c, Supplementary Figure 4). Enriched pathways were analysed for overall upregulated genes in IDTCs and perceived as a response to type 1 IFN (GO: 0034340), type 1 interferon signalling pathway (GO:0060337), cellular response to type 1 IFN (GO:0071357), and innate immune response (GO:0045087) (Supplementary Figure 5 and Supplementary Figure 6). This suggested that in response to targeted therapy and chemotherapy, cancer cells survive at an early state of stress-induced drug tolerance by increasing interferon signalling maintaining a slow proliferative state.

### Paucity of changes in global DNA methylation characterizes IDTCs

We then investigated DNA methylation as one of the plausible mechanisms of the repressive state in IDTCs. DNA methylation is reported to drive acquired drug resistance [21] and to interact with repressive histone marks like H3K9me3 in different cancer types [22]. In addition, we detected differential expression of three DNA methyltransferases DNMT3A, DNMT3B, and DNMT1 in IDTCs compared to parental cells, suggesting potential re-distributions of DNA methylation patterns in IDTCs (Supplementary Figure 7a). Genome-wide DNA methylation analyses were performed in biological triplicates using the HM450K BeadChip in four IDTC models (WM1366, WM164, A549, and HT29). Parental DNA methylation patterns were significantly different among the four cancer types ranging from a high global DNA methylation level in HT29 (mean β value = 0.62) to a low global DNA methylation level in WM164 (mean β value = 0.49; Supplementary Figure 7b). Particularly, we observed that global DNA methylation changes in each cell type followed different trends independent of the initial DNA methylation level. Thus, while IDTC HT29 showed substantial global hypo-methylation, IDTCs A549 and WM1366 presented moderate hyper-methylation and IDTC WM164 a substantial global hyper-methylation (Figure 3d).We then analysed the genomic context of the differentially methylated CpG sites for all pairs and found that, independently of the cancer type, the most significantly affected genomic regions were low CpG density segments named “Open Sea” (Supplementary Figure 7c). Mainly due to the substantial differences in intrinsic DNA methylation patterns among the four cell lines no common significantly differentially methylated CpG sites were found (adjusted *p*-value < 0.05) when comparing parental to IDTCs (Figure 3e). However, when comparing methylation profiles of IDTCs and their respective parental cells, we found significant differentially methylated regions (adjusted *p*-value < 0.05) suggesting that DNA methylation re-distribution in IDTCs is not a generalized mechanism, but a cell-type specific response to sustained drug exposure (Figure 3f).

### Genome-wide redistribution of histone marks defines IDTCs

**Figure 4:**
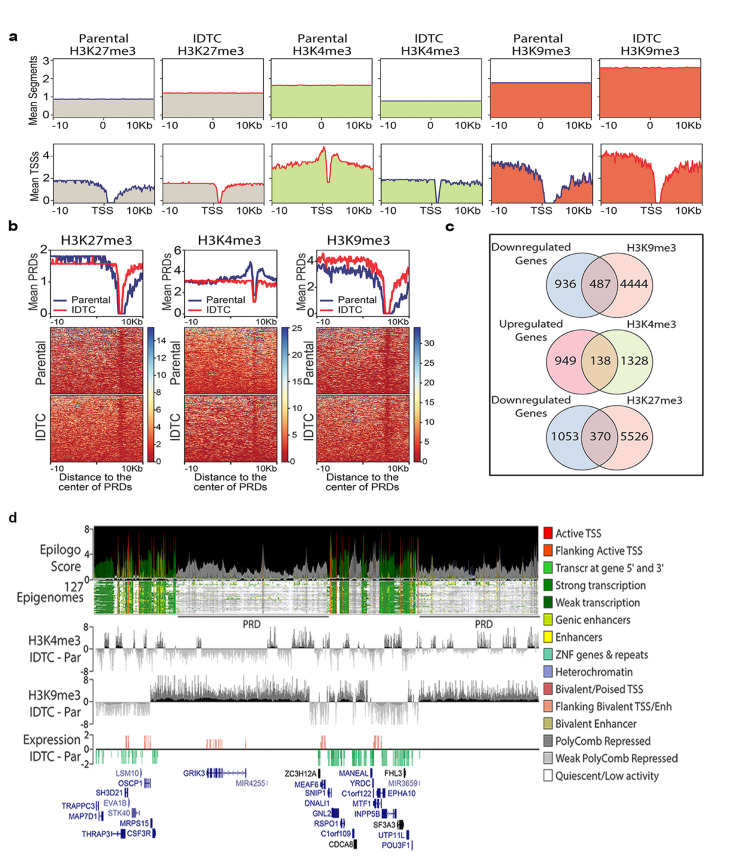
Genome-wide re-distribution of histone modifications in IDTCs **a.** Genome-wide levels of histone modifications are represented by the mean number of segments with ChIP-seq peaks 20kb around 100,000 randomly selected genome segments (upper panel). The association with transcriptomic changes is represented as the distribution of each histone modification around the transcription start sites (TSSs) of 1,423 genes down-regulated (fold change <-2) in IDTC cells (lower panels). **b.** Re-distribution of histone modifications in IDTCs was evaluated on 1,245 polycomb repressive domains (PRDs) associated with IDTC down-regulated genes. **c.** A significant overlap was observed between down-regulated genes (*n* = 1,423) and genes associated with H3K9me3 (*n* = 4,969)(*n* = 487; exact hypergeometric probability; *P* = 5.38x 10^-22^) as well as for regions between up-regulated genes (fold change >2; *n* = 1,087) and genes associated with H3K4me3 (*n* = 1,487)(*n* = 138; exact hypergeometric probability; *P* = 3.15x10^-12^) and between downregulated genes (*n* = 1423) and genes associated with H3K27me3 (*n* = 370; exact hypergeometric probability *P* = 0.03). **d.** Representative genomic view of the inversely correlated H3K4me3 and H3K9me3 modifications in IDTCs. The chromatin states were identified by calculating the Epilogos score (https://epilogos.altiusinstitute.org/) from the integration of the Core 15-state model using 111 reference human epigenomes generated by the Roadmap Epigenomics Project [23] plus 16 human epigenomes generated by the ENCODE Project. Two PRDs showing enrichment in H3K9me3 and depletion of H3K4me3 leading to a regional (2.6 Mb window) transcriptional down-regulation in IDTCs.

The characteristic changes of histone marks in IDTCs prompted us to further investigate the whole-genome distribution of H3K4me3, H3K27me3 and H3K9me3 modifications in WM164 IDTCs compared to untreated control by ChIP-seq. Overall, an increased level of H3K9me3 decreased level of H3K4me3 and relatively unchanged level of H3K27me3 was observed in IDTCs (Figure 4a, upper panel). To evaluate the influence of histone modifications on gene expression regulation, we evaluated changes in the genomic vicinity of IDTC down-regulated genes (fold change -2; *n* = 1,423). In concordance with repressive epigenomic reprogramming, a characteristic peak distribution of H3K4me3 on transcriptionally active genes was observed in paternal cells which decreased around the distal (±1.5kb) transcription start sites (TSSs) in IDTC cells. Additionally, the level of H3K9me3 was increased in distal promoter region (>5kb away from the TSSs) and proximal regulatory elements of negatively-regulated genes in IDTCs (Figure 4a, lower panel). Moreover, we identified that 1,245 of the 1,461 down-regulated genes were associated with polycomb repressive domains (PRDs). Interestingly, we observed a gain of H3K9me3 levels in these regions which might be associated with transcriptional repression of these genes (Figure 4b). Overall, we observed significant overlapping between down-regulated genes and gain on H3K9me3 (*n* = 487; *P* = 5.38x 10^-22^), between up-regulated genes and gain on H3K4me3 histone marks (*n* = 138; *P* = 3.15x10^-12^) and down-regulated genes with repressive H3K27me3 mark (*n* = 370. *P* = 0.03, Figure 4c). To further understand the genomic context of the regions exhibiting changes on histone modifications in drug tolerance, we integrated our data with the core 15 states chromatin maps generated from 127 human epigenomes [23]. Genomic regions with concomitant gains in H3K9me3 and loss of H3K4me3 defined domains of low transcriptional activity significantly overlapped with PRDs (Figure 4d). This suggests that concordant modulation of active and repressive histone marks may lead to transcriptional activation or repression in IDTCs.

### Increased H3K9me3 is characterized by dynamic expression of histone modifiers

**Figure 5:**
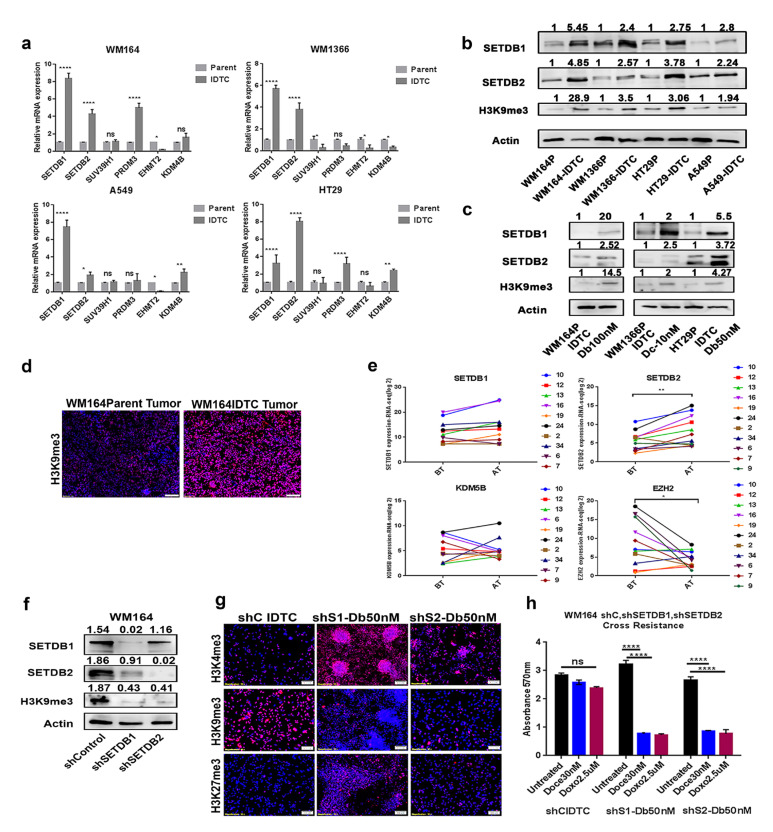
SETDB1 and SETDB2 regulate H3K9me3 in IDTCs and knockdown restores drug sensitivity **a.** RNA was isolated from different IDTCs and their parental cells. q-RTPCR was performed for the different histone modifiers of H3K9me3. Actin RNA was used as an internal control and relative gene expression levels were calculated as delta CT. Bars represent the mean of three biological replicates. Statistical analysis was performed by two-way ANOVA test and P-value is represented as (*) where, ****P < 0.0001, ***P < 0.001, **P < 0.01, and *P < 0.05. **b.** Proteins were isolated from different IDTCs and their respective parental cells. Immunoblots were performed to investigate the expression of SETDB1, SETDB2, and H3K9me3. **c.** WM164, WM1366, and HT29 cells were exposed to 100nM dabrafenib, 10nM docetaxel and 100nM dabrafenib respectively for two weeks. Proteins were isolated from IDTCs and untreated control and immunoblotted for SETDB1, SETDB2 and H3K9me3. All western blot images were quantified by ImageJ software. Values were normalized by subtracting from loading control. **d.** Tumour tissue was formalin fixed and tissue slides were stained for H3K9me3. Images were taken in a Vectra III Spectral scanner. **e.** RNA-sequencing data of paired pre- and post-treatment tumour biopsies derived from melanoma patients from GEO [55] were normalized, background-corrected and analysed using the R package “lumi”. Statistical analyses were performed by paired t-test where, **P < 0.01, *P < 0.05. Each paired match-patient expression value for an individual gene is shown by different colours. Numeric values indicate the patient number as provided in Supplementary Table 2. BT- Before treatment, AT- After treatment. **f.** Lentiviral transduction was performed according to manufacturer protocol (Sigma Aldrich, MA, USA). Protein lysates of the transduced cells were subjected to immunoblotting for SETDB1, SETDB2, H3K9me3 and Actin. **g.** WM164 shcontrol, shSETDB1, and shSETDB2 transduced cells were exposed for 16 days to 50nM dabrafenib and stained for H3K4me3, H3K9me3, and H3K27me3 by immunofluorescence. **h.** Same as in (g) but in addition challenged with toxic concentrations of docetaxel (30nM) and doxorubicin (2.5µM). Cell survival was analysed by MTT assay. Statistical analysis was performed by two-way ANOVA test and P-value is represented as (*) where, ****P < 0.0001. All western blot images were quantified by ImageJ software. Values were normalized by subtracting from loading control.

The increase in H3K9me3 prompted us to test for different known histone modifiers. H3K9me3 is catalysed by a set of specific histone methyltransferases and demethylases [24]. Our microarray data suggested the reciprocal up-regulation of methyltransferases and down-regulation of demethylases specific for H3K9me3 (Supplementary Table 2). Probing for SETDB1, SETDB2, EHMT2, SUV39H1, PRDM3, and KDM4B using qRT-PCR in WM164, WM1366, A549, and HT29 showed a consistent upregulation of SETDB1, SETDB2 and inconsistent changes in the expression of other histone modifiers in all IDTCs compared to their parental cells (Figure 5a). Higher expression of SETDB1, SETDB2 and H3K9me3 was confirmed at the protein level in four IDTC models representative of melanoma, lung and colon cancer (Figure 5b). Additionally, chronic exposure to dabrafenib and docetaxel at high concentrations led to a similar transition to the IDTC state involving SETDB1, SETDB2 and H3K9me3 up-regulation (Figure 5c). However, short exposure to dabrafenib, docetaxel, and doxorubicin for 72 hours did not induce SETDB1/2 and H3K9me3 which proposes that these changes are indeed features of the IDTC state (Supplementary Figure 8a). These observations suggest that increased expression of histone methyltransferases SETDB1 and SETDB2 stabilize H3K9me3 which is characteristic across different cancer types as a generic response towards stress. This *in vitro* increase of H3K9me3 was further confirmed in a melanoma xenograft model. Tumours generated by injecting 1x10^6^ WM164 melanoma cells and treated with dabrafenib (10mg/Kg) and trametinib (0.1mg/Kg) after formation of tumours (200mm^3^) for 21 days, resembling the *in vitro* observations, showed an increase of H3K9me3 compared to control (Figure 5d, Supplementary Figure 8b). For further validation paired clinical specimens from melanoma patients treated with targeted therapies were analysed (Supplementary Table 3). RNA-seq data showed a significant up-regulation of SETDB2 after therapy and a decrease in EZH2, a methyltransferase of H3K27me3, with a trend towards increased SETDB1. KDM5B was either up- or downregulated (Figure 5e).

### Silencing of SETDB1 and SETDB2 restores drug sensitivity

Knockdowns of SETDB1 or SETDB2 lead to a significant reduction of H3K9me3 (Figure 5f). Interestingly, knockdown inhibited the acquisition of induced drug tolerance. This was evidenced by loss of H3K9me3 but gain of H3K4me3 and H3K27me3 compared to shcontrol cells indicating a switch of the characteristic expression of histone marks found in IDTCs. shSETDB1 cells exposed to dabrafenib gained a preferential growth advantage by forming cellular clusters (Figure 5g). shcontrol IDTC, shSETDB1 and shSETDB2 cells were further exposed to high concentrations of docetaxel (30nM) or doxorubicin (2.5µM) which renders the sensitivity of shSETDB1/2 treated cells (*P* < 0.0001) but not shcontrol IDTC (Figure 5f). A similar effect was observed in shSETDB1/2 A549 cells compared to shcontrol (Supplementary Figure 8c-8e).

## DISCUSSION

**Figure 6:**
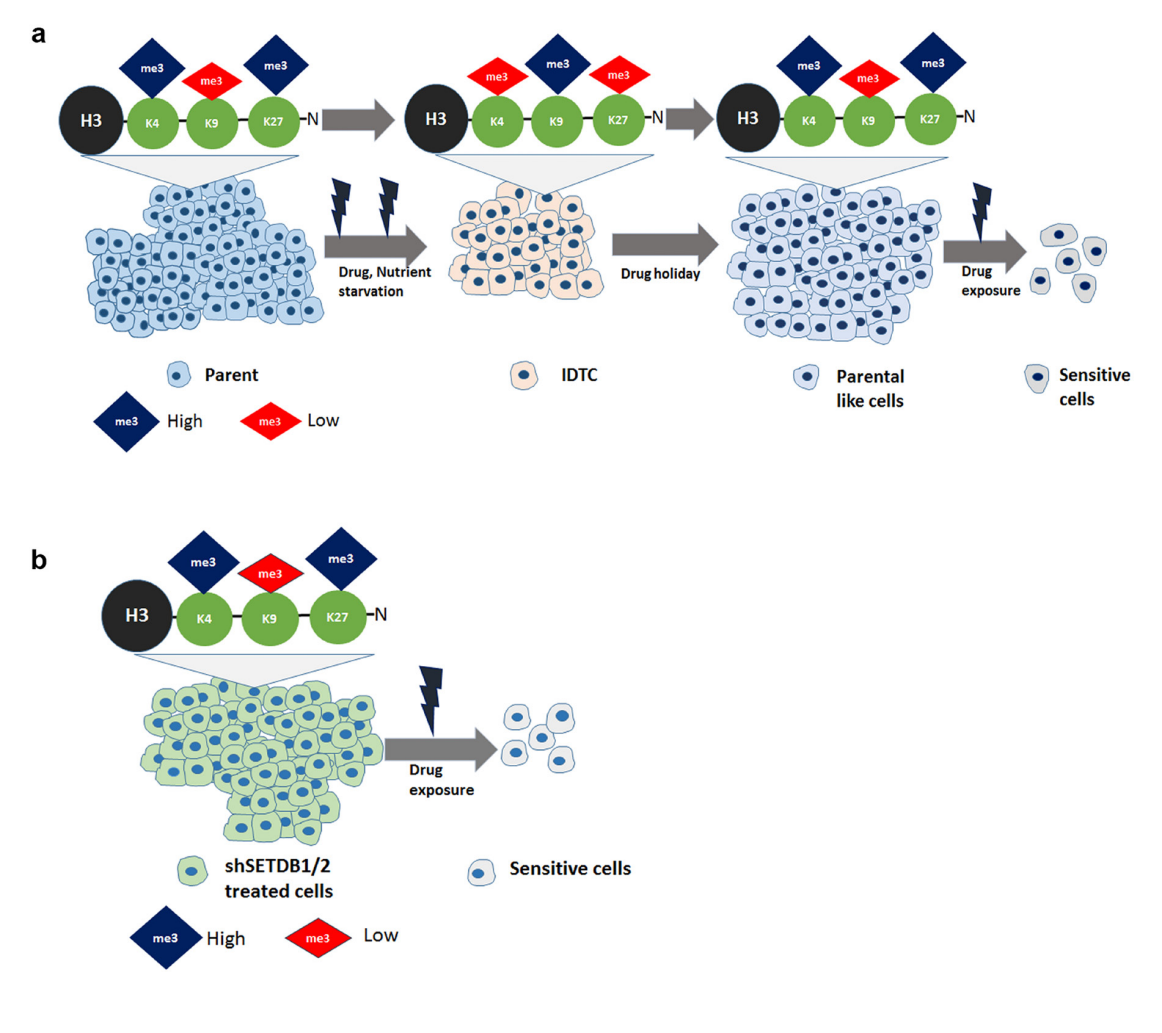
Model **(a)** Stress induce cancer cells to undergo a dynamic histone reprogramming with the increase of repressive histone mark H3K9me3 and decrease of active mark H3K4me3 and repressive mark H3K27me3. These distinct histone modifications help cancer cells to maintain a transcriptionally repressed slow cycling state. Drug holiday reversed the histone modifications similar to that of parental cells which induce sensitivity. **b.** Upon drug treatment silenced SETDB1/2 cancer cells reprogram their histone modifications which are sensitive to other drugs.

Cancer cells exert an innate response towards different stressors transforming them into a stress-resistant slow proliferative state by histone remodelling. These induced drug-tolerant cells (IDTCs) exhibit cross-resistance to much higher concentrations of the same or other drugs [12]. IDTCs are characterised by the upregulation of CD271, multidrug resistance and increased expression of major signalling pathways such as the MAPK (Raf/Ras/MEK/ERK) and the PI3K/AKT/mTOR. Histone remodelling is characteristic of IDTCs along with alterations of signalling cascades during this transformation. We have found that the transcriptional active mark H3K4me3 and the repressive mark H3K27me3 decreased, whereas a repressive mark H3K9me3 increased at the IDTC state compared to parental cells (Figure 6a). This is in line with the previous finding of an increase of KDM5B regulating H3K4me3 and loss of H3K27me3 which was associated with poor prognosis and drug resistance in different cancer types [25-27]. Previous studies indicated that H3K9me3 correlates with tumour suppressor gene silencing and the increase of H3K9me3 being a strong predictor of poor survival in different cancer types [28-30]. We found that accumulation of the repressive mark H3K9me3 along with loss of the active mark H3K4me3 is associated with the ability of cancer cells to survive early stress. The continued survival of these cells in the presence of drug allows these cells to establish over time irreversible drug resistance mechanisms. Employing a timely drug holiday causes epigenetic reprogramming to a similar histone methylation pattern as parental cells. Cancer cells suffer a fitness deficit upon drug withdrawal which renders them again sensitive to the drug (Figure 6a), a phenomenon recently described [11]. A previous study denotes that drug holiday-induced sensitivity towards the same therapy is due to an oncogene-induced senescence-like state by supra-physiological levels of BRAF-MEK-ERK1/2 signalling in melanoma [31]. Along this line it was reported that ERK2 binding to promoter regions induces the H3K4me3 mark in human embryonic stem cells [32]. However, the detailed underlying mechanism of regulating histone modifications upon drug holiday requires further investigation. This suggests that cancer cells exhibit a generic response to stress at the initial stage but can reverse their epigenetic makeup depending on the context demonstrating phenotypic plasticity in cancer [33]. Our study shows that IDTCs have a reduced global gene expression level compared to their respective parental cells and suggests that the IFN-α, -β, -γ signalling and cytokine signalling pathways are crucial for all IDTCs. This is supported by recent findings of constitutive activation of STAT/IFN signalling linked to drug resistance in different cancers [34, 35]. Other studies report that methyltransferases of H3K9 such as SETDB1 and SUV39H1 interact with DNA methyltransferases DNMT3B and DNMT3A, respectively, providing a functional link between DNA methylation and H3K9me3 modification [22, 36]. We found that there were no significant changes of global CpG methylation common to all IDTCs, suggesting that DNA methylation changes follow a cancer type-specific program. Our data demonstrate that IDTCs from different cancer types exhibit a specific DNA methylation reprogramming that might not be generalized as a mechanism for the acquisition of drug tolerance. This is in line with an earlier study where it has been reported that deletion of DNMTs has no effect on H3K9me3 marking of pericentromeric heterochromatin in mice suggesting that DNA methylation at genic promoters is only weakly correlated with H3K9me2 or H3K9me3 in somatic cells and mouse embryonic stem cells [37, 38]. In parallel, another study demonstrated that SETDB1/H3K9me3 and DNMTs mark and regulate a distinct set of genes and retroelements in mESC [39]. Altogether, these findings suggest that histone modifications can act independently of DNA methyltransferases (DNMTs) repressing a target set of genes.

Whole-genome redistributions of H3K4me3 and H3K9me3 suggest that due to the global decrease of the active histone mark H3K4me3 and the regional increase of H3K9me3 a subset of genes are transcriptionally suppressed in IDTCs. This is in line with previous findings claiming that genomic regions with loss of the active histone mark H3K4me3 but gain of the repressive mark H3K9me3 are associated with down-regulation of genes [40]. Here we show to the best of our knowledge for the first time dynamics in histone modifications which are associated with transcriptional repression at early drug resistance in cancer. ChIP-microarray overlay also suggests that polycomb repressor domains are downregulated due to the increase in H3K9me3 and decrease of H3K4me3 mark which supports previous findings on the association of drug resistance in cancer with the concomitant loss of the components of polycomb repressor complex [41, 42]. This implies that the interplay amongst active and repressive histone modifications are decisive to regulate gene expression in response to drugs or other stressors in cancer cells.

Previously it has been shown that stable knockout of KDM5B reversed the slow cycling population in melanoma cells [43]. In our previous study, stable knockdown of KDM5B restored drug sensitivity the reminiscent population, however, exhibited drug resistance against single or combinatorial treatments resembling IDTCs [12]. However, stable knockdown of SETDB1/2 in this study reversed early acquired drug resistance with dynamic re-enhancement of the active mark H3K4me3 (Figure 6b). The underlying mechanisms of histone modifications are poorly understood although some studies demonstrated the role of signalling cascades in regulating histone modifiers in a context-dependent manner. Insulin growth factor 1 receptor (IGF1R) was found to be a direct regulator of KDM5A in a drug-tolerant model. Inhibition of IGF1R ablated the drug-resistant state by downregulating KDM5A resulting in an increase of the H3K4me3 mark [6]. Another study revealed that activated AKT phosphorylates EZH2 and BM1 resulting in inhibition of H3K27 methylation by suppressing the methyl transferase activity of EZH2 [44]. Further type1 interferon (IFN1) signalling was reported to induce SETDB2 mediated H3K9me3 marking on several antiviral effector genes resulting in transcriptional repression upon influenza A virus infection in a murine and human macrophage model [45]. Indeed, we have observed a consistent activation of PI3K/AKT and IFN1/STAT1 signalling which might play a role in the concomitant modulation of the histone marks. Along this line, our previous study revealed that combined inhibition by using PI3K/AKT, IGF1R inhibitors along with the primary drug eliminates the IDTC transition from parental cells, however, IDTCs displayed tolerance once they are exposed to combined inhibitors [12]. Therefore, we propose that stress-induced multiple signalling cascades might play a crucial role in the acquisition and regulation of distinct histone modifications leading to global transcriptional downregulation. Our findings reveal generic changes of histone modifications in cancer upon drug exposure and nutrient starvation. This also implies that a defined epigenetic modification is pivotal in IDTCs to maintain a slow proliferative state in response to stress and targeting histone modifiers might prevent the IDTC transition from parental cells.

This study focused on the early time point of acquired drug resistance in cancer, therefore SETDB1/2 mediated increment of H3K9me3 mark along with concomitant loss of H3K4me3 is limited to the early phase of stress tolerance in cancer. It will be important to investigate the role of distinct histone remodelling in acquired drug resistance with a prolonged time point which might simulate the relapse of cancer patients.

In the future, it will be crucial to underpin the role of upstream signalling cascades such as IFN and cytokine signalling involved in the distinct modification of histone marks from parental to IDTCs. A more detailed understanding of early drug tolerance will allow identification of potential targets as epigenetic ‘drivers’ of this process which implemented in addition to established treatments might prevent the transition to permanent resistance.

## MATERIALS AND METHODS

### Antibodies and reagents

CD271, SETDB1, SETDB2, p-mTOR and Actin antibodies were purchased from Abcam, (CBG, UK). Phosphorylated AKT-Ser473 (pAKT), total AKT (T-AKT), phosphorylated ERK1,2-Thr202/Tyr204 (p-ERK1,2), total ERK (ERK1,2) antibodies were obtained from Cell Signalling Technology, (MA, USA). Antibodies for H3K4me3, H3K9me3 and H3K27me3 were purchased from Active Motif (CA, USA). Alexa Fluor 594 antibody and Hoechst 33342 were obtained from Thermo Fisher Scientific (MA, USA).

### Drugs

Chemotherapeutic drugs doxorubicin, docetaxel, and cisplatin and targeted inhibitors such as dabrafenib, trametinib, erlotinib were purchased from Selleck Biochem, TX, USA.

### Cell lines

Melanoma cell lines WM164, WM1366 were obtained from The Wistar Institute. Lung cancer cell line A549, colon cancer cell line HT29 and hepatocellular carcinoma cell line HEPG2 were kindly provided by Dr Gerald Hoefler from the Medical University of Graz, Austria. HCC827 cell line was kindly provided by Dr Derek Richard, The University of Queensland, Brisbane, QLD. The SKBR3 cell line was kindly provided by Dr Fiona Simpson, UQDI, The University of Queensland, Brisbane, QLD. Cell lines are authenticated by STR profiling and tested for mycoplasma contamination

### Cell culture

Cells were cultured in RPMI media (Life technology, USA) supplemented with 5% heat-inactivated fetal bovine serum (FBS, Sigma, MI), 10% of L-Glutamine and 10% of penicillin-streptomycin antibiotics (Life Technologies, CA).

### Drug treatment and nutrient starvation

Cancer cells were exposed to either DMSO or sub-lethal dosages of different drugs such as dabrafenib, docetaxel, doxorubicin and erlotinib for 12-15 days for the generation of IDTCs. For nutrient starvation cancer cells were cultured with 1mg/ml glucose containing media for at least 12 days. Media and drugs were replenished every three days.

### Drug holiday

Cancer cells were continuously exposed to various stresses (different drugs, nutrient starvation) for 12-15 days for generating IDTCs. After formation of IDTCs, the drug was withdrawn and cells kept in culture for another 10 days as suggested based on clinical trials [46]. Media was replenished every three days.

### Crystal violet staining

IDTCs and their parental cells were washed with 1XPBS and then fixed with 4% paraformaldehyde, followed by 30 min incubation. Fixed cells were incubated with 0.05% crystal violet solution in 1xPBS for one hour. The plates were then washed three times with reverse osmosis (RO) H_2_O and pictures were taken using the Bio Rad gel documentation unit after drying.

### Cell survival assay

Cell survival was analysed by MTT assay. Briefly, 2x10^4^ cells were seeded in 96-well culture plates. On the following day cells were exposed to the drugs as indicated above and after 72 hrs of drug exposure cells were incubated with MTT (3-(4, 5-dimethylthiazolyl-2)-2,5 diphenyltetrazolium bromide) reagent (Life Technologies, CA) (10µl) at 37°C for 4hrs. 100µl detergent reagent was then added to the wells and incubated in the dark at room temperature for 4hrs. Absorbance was measured at 570nm using a microtiter plate reader.

### Cell lysates and immunoblots

Immunoblotting was performed as previously described [47]. Briefly, cell pellets were lysed with RIPA buffer for protein isolation. Protein samples were treated with protease and phosphatase inhibitor cocktail before immunoblotting. Protein concentrations were determined by BCA standard solution. Equal amounts of protein were loaded onto 10% SDS-PAGE gels. Polyvinylidene difluoride (PVDF) membrane was used for wet transfer. Transferred proteins were blocked with either 5% BSA (for phosphorylated proteins) or 5% milk in 1xTBST for one hour at room temperature. The membrane was then incubated with a specific primary antibody overnight at 4°C, then washed three times with 1xTBST and incubated with a secondary antibody at room temperature for 1hr followed by three washes with 1xTBST. The membrane was developed with ECL solution and image taken with LI-COR C-DiGiT western blot scanner.

### Immunofluorescence

Immunofluorescence(IF) analysis was performed as described previously [12]. Briefly, treated or untreated cells were fixed with 4% paraformaldehyde in PBS for 30minutes and then blocked with 0.3% Triton X-100, 5% goat serum and 1% BSA dissolved in PBS. Cells were incubated with the primary antibody at 4°C overnight; followed by three washes with 1% BSA in PBS on the following day. Cells were incubated with Alexa fluoro 594 secondary antibody for one hour along with Hoechst dye for the nuclear staining. All images were taken with an Olympus inverted fluorescent microscope.

### RNA isolation and q-RTPCR

Total RNA was isolated and cDNA was synthesized according to the manufacturer’s protocol (Bioline, MA, USA cat. BIO-65043). The SensiFast SYBR Lo-ROX master mix from Bioline was used to amplify the specific genes and products were detected by the VIIA-7 machine of Applied Biosystem. The following primers were used: SETDB1 for 5’- GCTGAGACACCAAACGTCAAAA-3’, rev 5’-ACATAGGAAGCATAGCCATCATCA-3’, SETDB2 for 5’-GAGTCTGCAGAACGGCATC-3’,rev 5’-CACGCCCACATCACTGTAGA-3’, SUV39H1 for 5’-GTCATGGAGTACGTGGGAGAG-3’, rev 5’-CCTGACGGTCGTAGATCTGG-3’, G9a for 5’-TGGGAAAGGTGACCTCAGAT-3’ , rev 5’-TCCCTGACTCC TCATCTTCC-3’, PRDM3 for 5’-GAAAATGGTAAAATGTTCAAAGACA-3’, rev 5’-CACCAGTCCTGTTGAACCAA-3’, KDM4B for 5’-GACATCAGCGGCTCTTTGTATGATG-3’,rev 5’-ATGCCGAAGTACAGGTAGGGCGTG-3’, Actin for 5’-CCACACTGTGCCCATCTACG-3’, rev 5’-AGGATCTTCATGAGGTAGTCAGTCAG-3’.

### Lentiviral transduction

Lentiviral transduction was performed according to manufacturer protocol (Sigma Aldrich, MA, USA). Briefly, WM164 cells were seeded and on the following day media were changed to polybrene (8ug/ml) containing media and incubated with lentiviral particles which were further selected for 2 weeks with 5µg/ml puromycin. Cells were transduced with lentiviral particles of non-target shRNA control (SHC016V, Sigma, MA), SETDB1 target shRNA (SHCLNV-NM_012432) and SETDB2 target shRNA (SHCLNV-NM_031915).

### *In-vivo* tumour staining

The frozen sections of harvested tumours were embedded in OCT (PST-IA018), QLD, Australia cut and transferred onto slides. The slides were fixed for 10 minutes in 4% paraformaldehyde and washed twice in PBS for 5 minutes each. The cells were permeabilized for 30 minutes in 0.2% Triton X and washed twice with PBS for 5 minutes each. 5% goat serum/1% BSA/0.2% Triton X 100 solution was used to block the slides for one hour. Slides were then incubated overnight at 4°c with the primary antibody diluted in blocking solution. Further, they were washed twice for 30 minutes in PBS. Slides were then incubated at room temperature with the respective secondary antibody in blocking solution for 2 hours, followed by 20 minutes in Hoechst nuclear stain and visualised under an Olympus IX73 microscope. The images were analysed by image J. All animal experiments were performed in accordance with institutional guidelines Ethics number: SOM/TRI/197/15/DRC.

### Microarray

Four cancer cell lines were analysed for genome-wide differential expression by Illumina expression array by the Australian Genome Research Facility (AGRF). These are WM1366 IDTCs (melanoma), A549 IDTCs (lung cancer), HT29 IDTCs (colon cancer), HCC827 IDTCs (EGFR mutant lung cancer) and their corresponding parental controls. The data was read into R using the Bioconductor package limma, 5, 6 and underwent normalization expression background correction using negative controls, quantile normalisation and then log2 transformed. The control probes were removed. All probes not expressed in the samples of interest were removed, resulting in 23,422 probes and 24 samples. A linear model was fitted contrasting the controls, resulting in all differentially expressed genes under a false discovery rate of 5%. The relative reliability of each array was estimated by measuring how well the expression values for that array follow the linear model.

### Genome-wide DNA methylation

Genome-wide DNA methylation assays were performed using the HM450K BeadChip (Illumina, Inc., Carlsbad, CA) platform as previously described [48]. The chips were scanned with Illumina iScan (Illumina, Inc.), and data was extracted using the R package methylumi. The ‘noob’ function in the R package minfi was used to process the data and then the ‘dasen’ function in the R package water melon for normalisation and dye-bias correction. DNA methylation levels were reported as β-value (β = intensity of the Methylated allele/(intensity of the Unmethylated allele + intensity of the Methylated allele)) and calculated using the signal intensity value for each CpG site.

### Chromatin immunoprecipitation (ChIP)

ChIP of WM164 IDTCs and parental cells were performed according to the manufacturer instructions (Active Motif, CHIP-IT Express Enzymatic Kit, Catalogue Numbers, 53009, 53035). Briefly, WM164 IDTCs and parental cells were formalin fixed for chromatin isolation. Isolated nuclei pellets were sonicated three times for 30s and then treated with an enzymatic shearing cocktail. 12 microgram of sheared chromatin was used for all samples for immunoprecipitation with the specific antibodies. ChIPed DNA concentration was measured in Qubit with high sensitivity dsDNA kit.

### ChIP-Sequencing

Chromatin immunoprecipitation sequencing (ChIP-Seq) was performed for H3K27me3, H3K4me3, and H3K9me3 on biological duplicates for paired parental and IDTC cells. ChIP-seq peaks were called as we previously described [49]. Briefly, raw sequence reads were mapped to the 1000 Genomes (b37) build of the human genome reference using BWA (version 0.7.5a) with default settings [50] and duplicates were marker using Picard Tools (version 1.103) (http://broadinstitute.github.io/picard/). Peaks were identified using the call peaks function in MACS2 [51] with a threshold set to -q = 0.01 and BigWig files were generated using bedGraphToBigWig [52]. BigWig files processing was performed using the deepTools webserver [53].

## SUPPLEMENTARY MATERIALS FIGURES AND TABLES


