# Supplementary material for: Distinct histone modifications denote early stress-induced drug tolerance in cancer
Source: Oncotarget. 2017 Dec 24;9(9):8206–22. doi: 10.18632/oncotarget.23654 (PMC5823586; doi:10.18632/oncotarget.23654)
Supplement: Supplementary file 1 [file oncotarget-09-8206-s001.pdf]

Distinct histone modifications denote early stress-induced drug tolerance in cancer

SUPPLEMENTARY MATERIALS

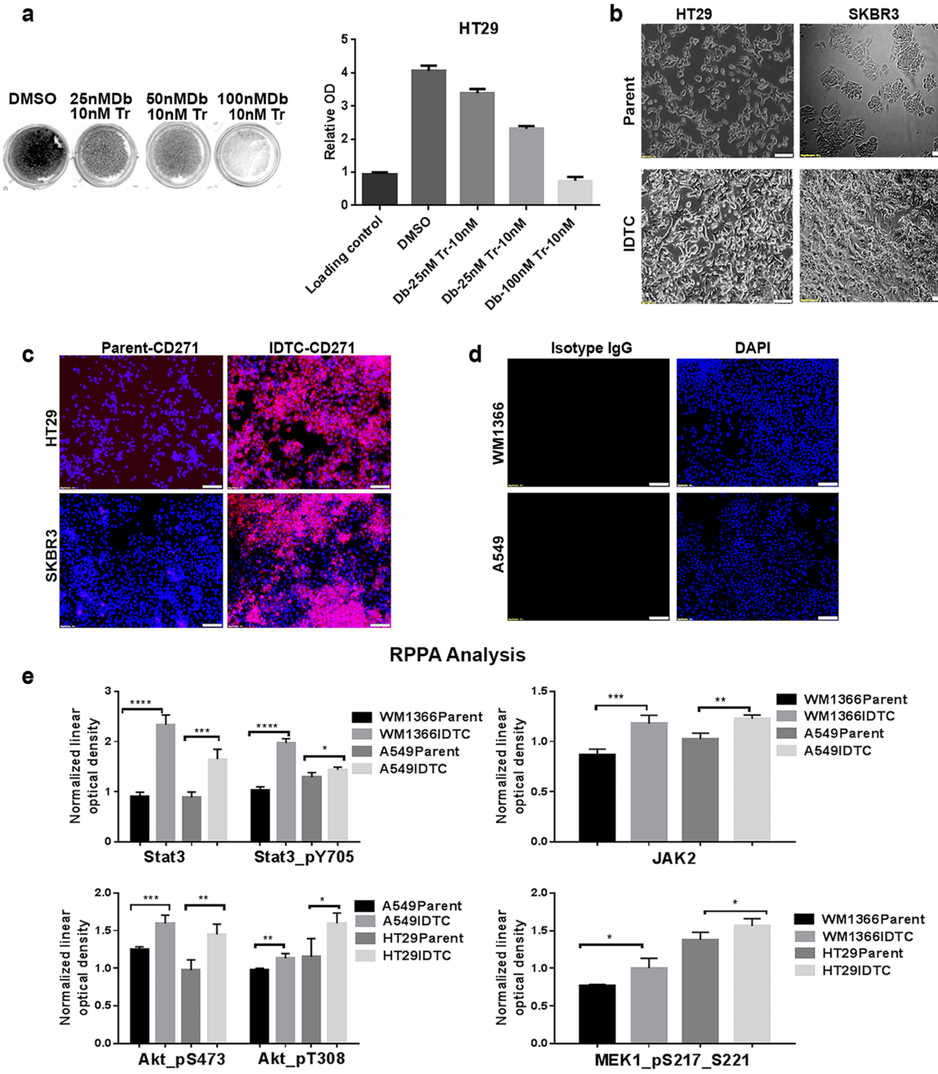

Supplementary Figure 1: Characterization of IDTCs (a) HT29 cells were exposed to 25nM dabrafenib and 10nM trametinib in triplicate for 12 days. Media with drug was changed every three days and DMSO was added to untreated control. After 12 days cells were stained with crystal violet staining in triplicate and one of them is shown as a representative image. **(b)** Distinct morphological feature of IDTCs compared to parent cancer cells. From bottom to top: HT29 IDTCs (Dabrafenib 25nM, Trametinib 10nM) compared to HT29 parent, SKBR3 IDTCs (Docetaxel 5nM) compared to parent. Bright field images were taken after 12 days of exposure to drugs for IDTCs (10x magnification). **(c)** CD271 expression was analysed by immunofluorescence in HT29 and SKBR3 IDTCs compared to their corresponding parental cancer cells. **(d)** Isotype control staining. WM1366 and A549 cancer cells were incubated with IgG isotype antibody and DAPI for nuclear staining and the expression of isotype control was analysed by immunofluorescence. **(e)** RPPA analysis described as normalized linear optical density value of proteins in WM1366, A549, and HT29 IDTCs and corresponding parent cells. All data points were normalized for protein loading and transformed to a linear value which is represented as bar graphs. Statistical analysis was performed by unpaired t-test and P-value is represented as (*) where, ****P≤0.0001, ***P≤0.001,**P≤0.01, *P≤0.05.

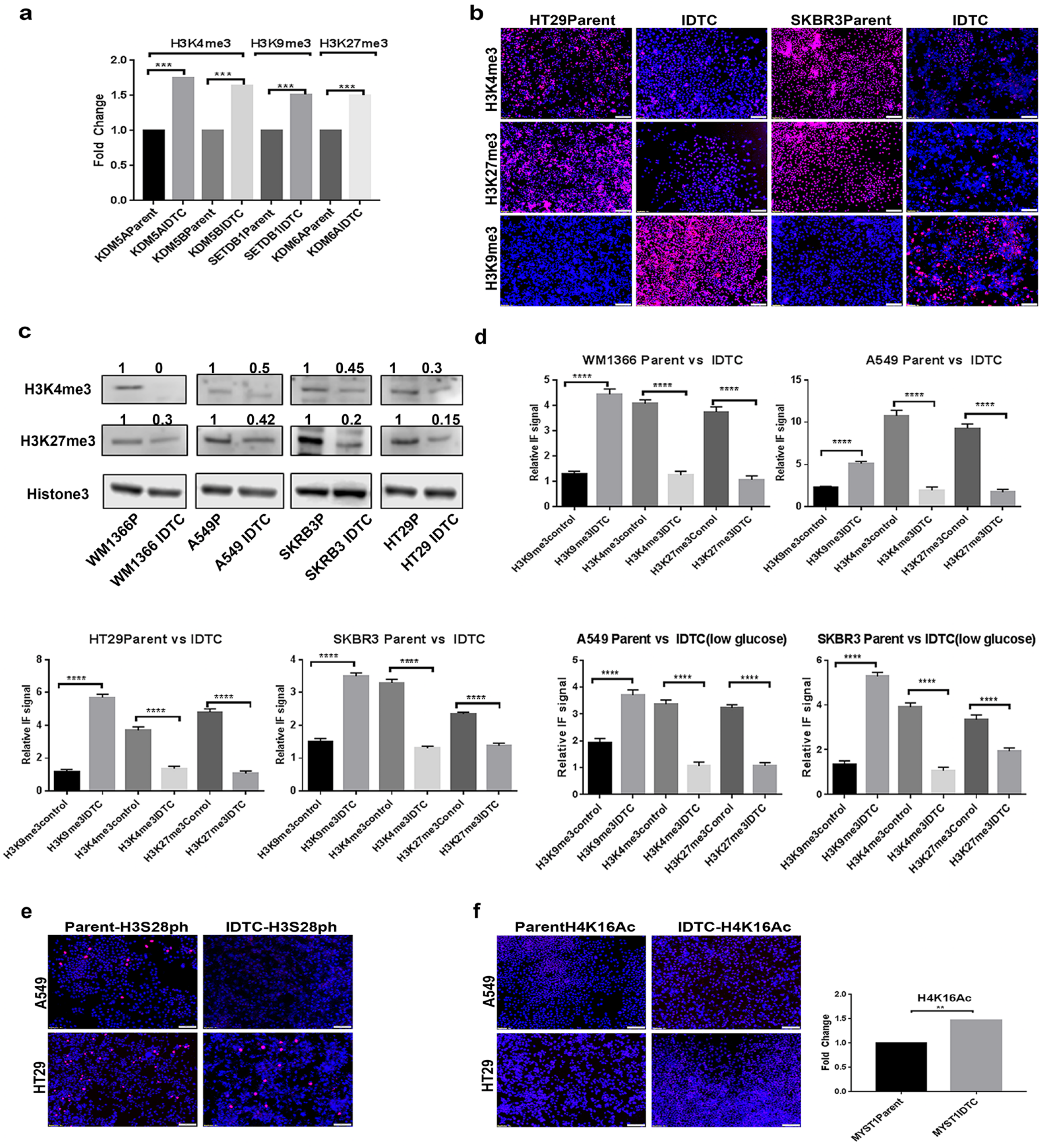

Supplementary Figure 2: Altered histone modifications in IDTCs compared to unexposed cells. **(a)** Expression value of WM164IDTC compared to parent WM164 from the previous microarray [1]. Fold change value are given on Y axis **(b)** IF staining of histone marks in different IDTCs. HT29 IDTCs (left panel), SKBR3 IDTC (right panel) compared to corresponding parent cells were stained with H3K4me3, H3K27me3 and H3K9me3. **(c)** Proteins were isolated from WM1366, A549, SKBR3, HT29 IDTCs and their corresponding parental cells lines. Proteins were immunoblotted for H3K4me3, H3K27me3. Histone 3 was used as loading control. All western blot images were quantified by ImageJ software. Values were normalized by subtracting from loading control **(d)**. Relative immunofluorescence (IF) signal of IDTCs compared to control. Overall signals of the IF were analysed in ImageJ software. Mean value of the three representative images was taken for plotting the bar graph. Unpaired t-test was performed for the analysis of statistical significance. P-value is represented as (*) where, ****P≤0.0001, ***P≤0.001,**P≤0.01, *P≤0.05. **(e, f)** Analyses of distinct histone marks by immunofluorescence. A549 and HT-29 IDTCs and their corresponding parental cells were analysed for pH3S28 (Abcam, CBG, 1:1000) and H4K16Ac (Cell Signalling Technology, MA, 1:500) histone modification by immunofluorescence. Differential expression of MYST1 in IDTC compared to control from previous microarray data [1].

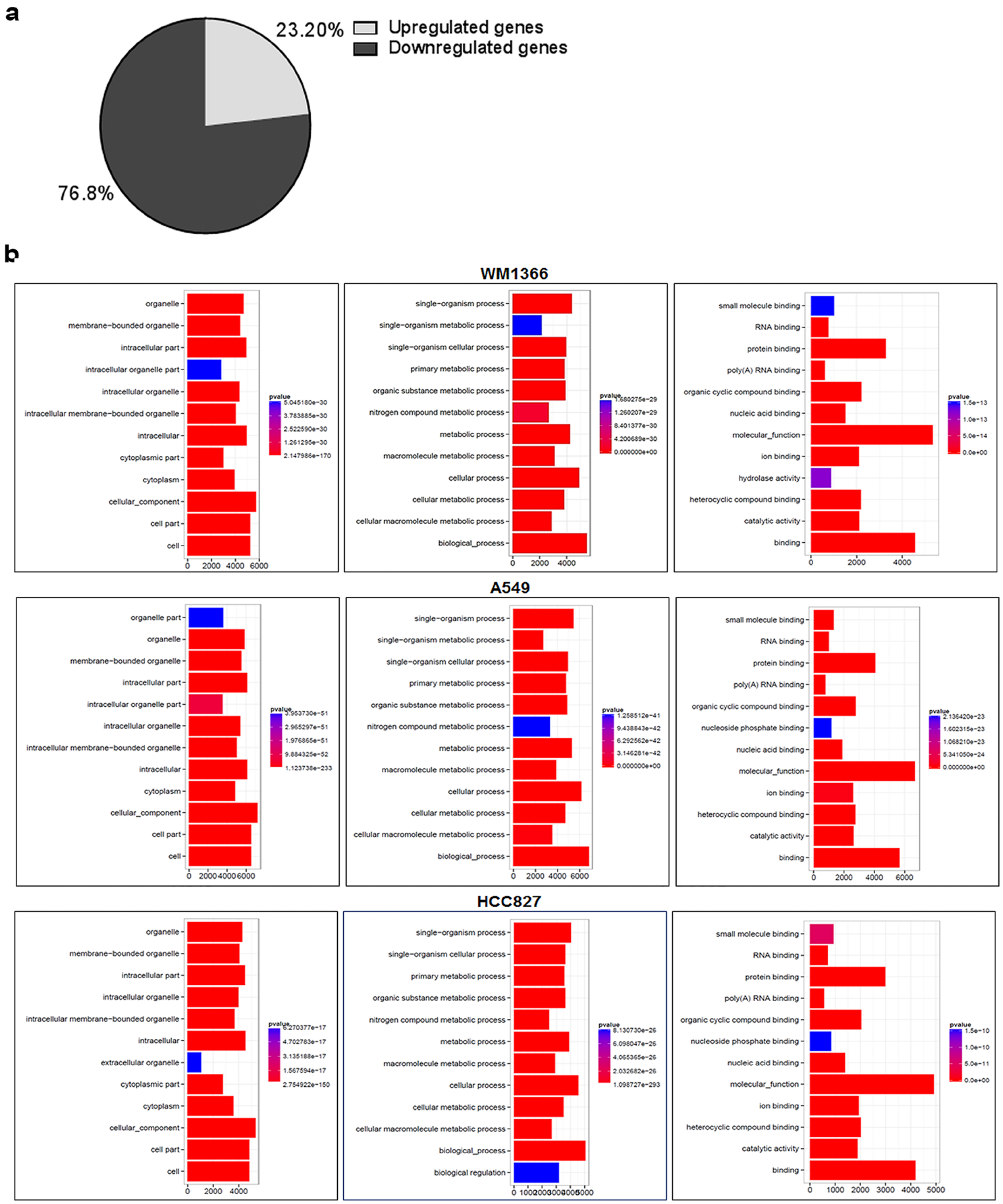

Supplementary Figure 3: Overall up and-down regulated genes and GO biological of different IDTCs (a) overall up and down regulated genes from cDNA microarrays. Pie diagram showing the percentage of up-regulated and down-regulated genes in IDTCs compared to untreated parent cells. Statistical analysis was performed using the Mann-Whitney unpaired t-test where P<=0.05 **(b)** GO enrichment of the IDTCs compared to parent: Gene Ontology for all three branches was undertaken using clusterProfiler7, using a p-value cut off of 0.1 and a q-value cut off of 0.05.

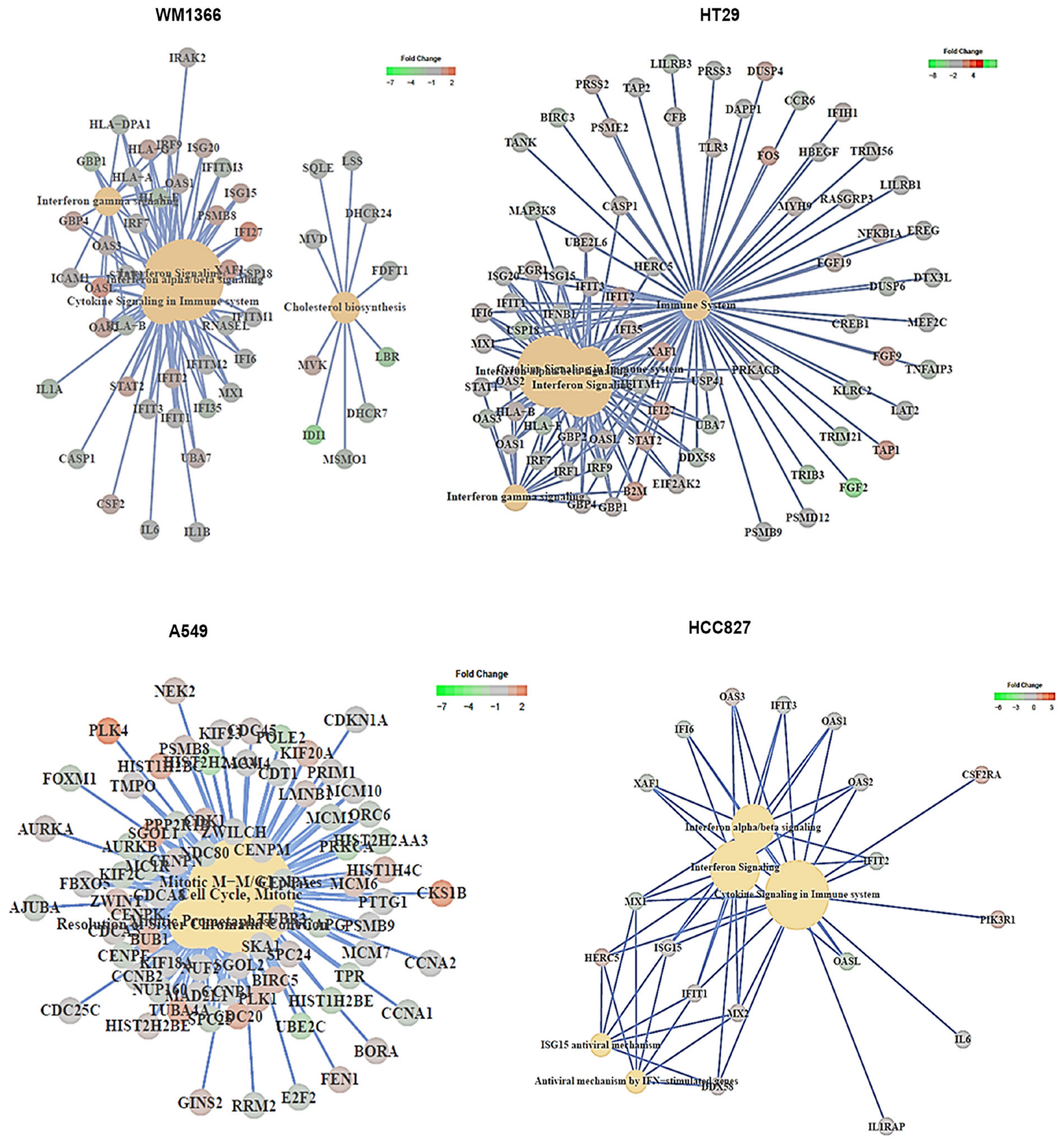

Supplementary Figure 4: Individual protein-protein interaction (PPI) networks of IDTCs. Enriched network shown for differential genes of IDTCs vs parental cells generated by Reactome. Fold change are shown with different colours in each set. See Supplementary Data Sheet in Supplementary Files for pathway enrichment gene list.

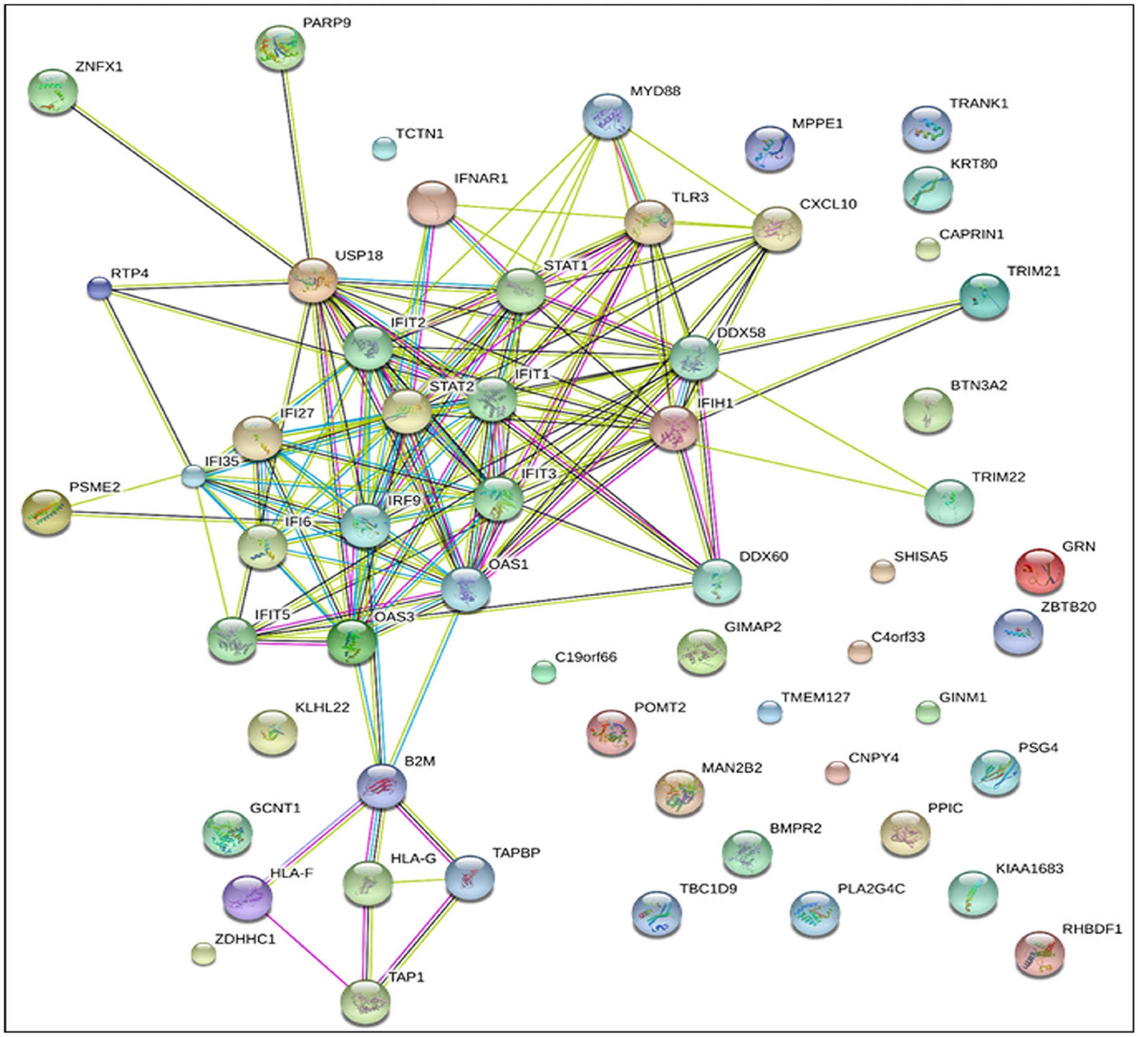

Pathway ID	Pathway description	Count in gene set	P- value
GO:0034340	Response to type I interferon	14	1.6e-19
GO:0060337	Type I interferon signalling pathway	14	1.6e-19
GO:0071357	Cellular response to interferon	14	1.6e-19
GO:0045087	Innate immune response	24	6.86e-15

Supplementary Figure 5: Overall protein-protein interaction (PPI) network. An enriched network was generated by string data base (http://string-db.org/) with all common up-regulated genes in four IDTC models and their pathways are shown as a table at the bottom.

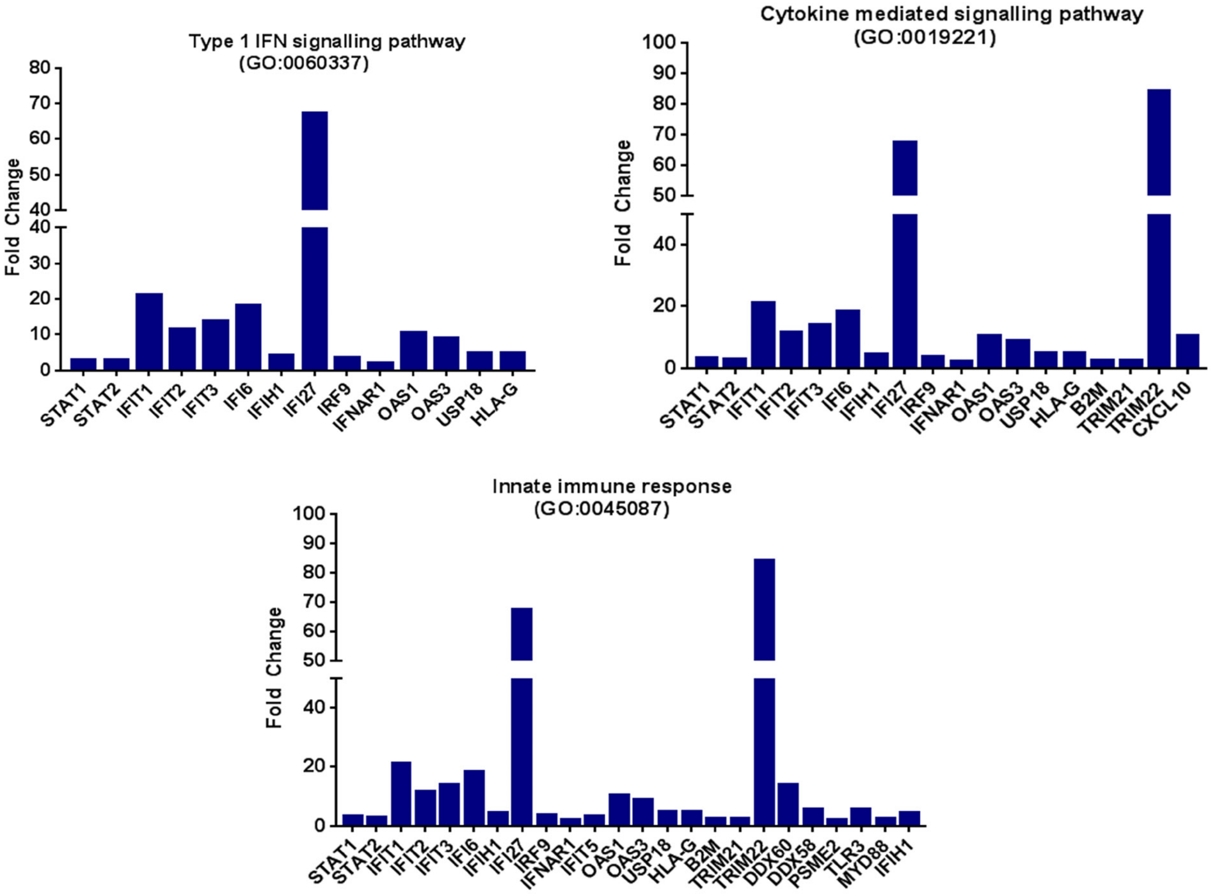

Supplementary Figure 6: Common upregulated genes and their fold change associated with enriched pathway: Fold changes are shown in Y axis and X axis represents the list of common genes associated with enriched pathway as shown in the table of supplementary Figure 5 (FC>1.5 and P<0.05).

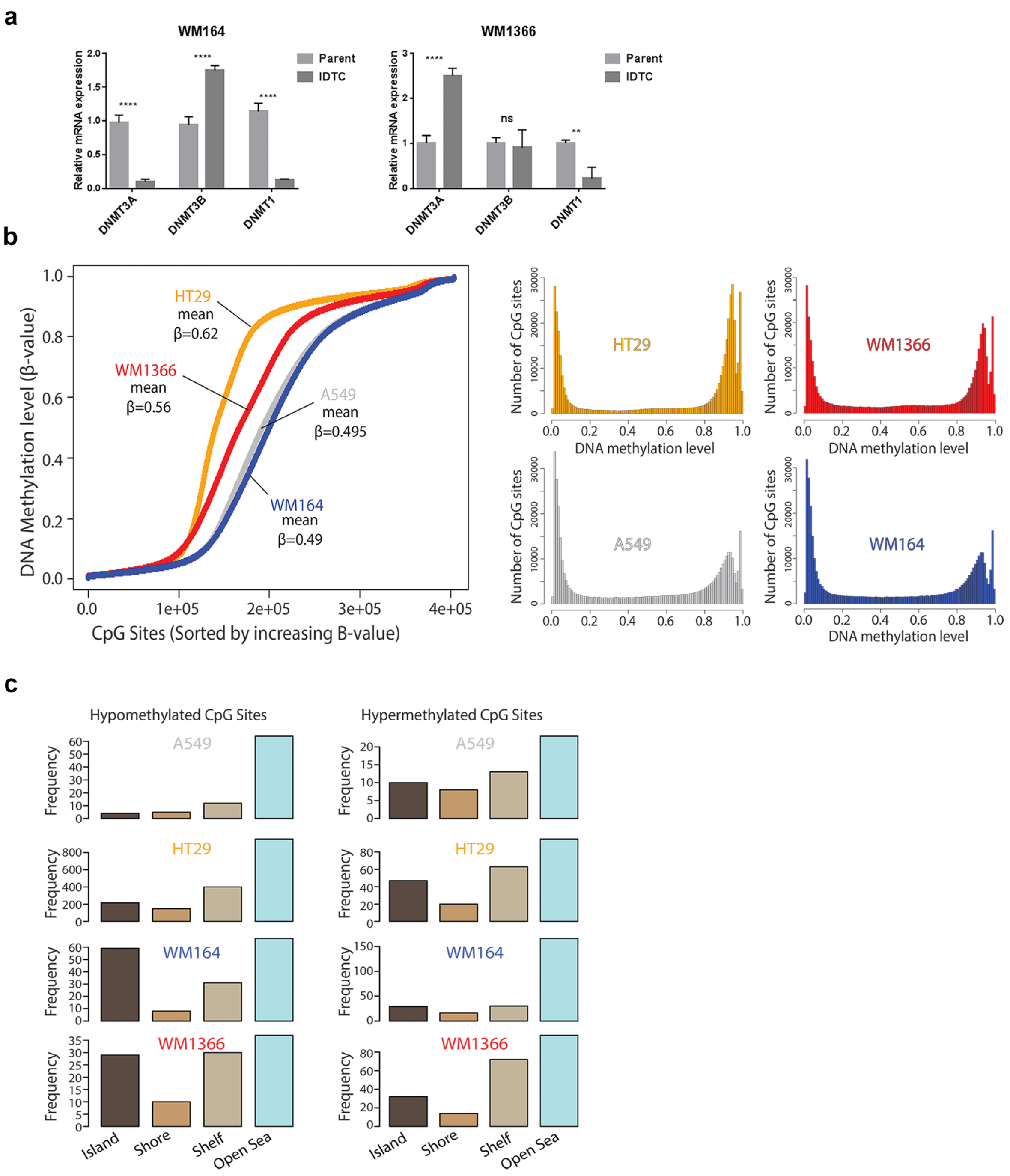

Supplementary Figure 7: Differential expression and dynamics of DNA methylation in IDTCs (a) Expression analysis of DNA methyltransferases by q-PCR. RNA isolation and cDNA synthesis was performed according to the manufacturer instruction as described in methodology. 36B4 was used as an internal control. The following primers were used DNMT3A for 5'-GACTCCATCACGGTGGGCATGG-3', rev 5'-TGTCCCTCTTGTCACTAACGCC-3', DNMT3B for 5'-GAGTCCATTGCTGTTGGAACCG-3', rev 5'-ATGTCCCTCTTGTCGCCAACCT-3',DNMT1 for 5'-GGTTTCCTTCCTCAGCTACTGCGA-3', rev 5'- CACTGATAGCCCATGCGGACCA-3'. 36B4 for 5'-GATTGGCTACCCAACTGTTGC-3', rev 5'- CAGGGGCAGCAGCCACAAAGG-3'.Statistical analysis was performed by two-way ANOVA test and P-value is represented as (*) where, ****P≤0.0001, ***P≤0.001, **P≤0.01, *P≤0.05. **(b)** Comparison of DNA methylation levels among parental cancer cell lines. Overall the global DNA methylation levels were compared among WM164, WM1366, A549 and HT29 untreated cells by sorting the CpG probes for increasing Beta values (left panel).The frequency distribution is represented by histograms as shown on the right panel. **(c)** Distribution of differentially methylated CpG sites according to CpG island, context.

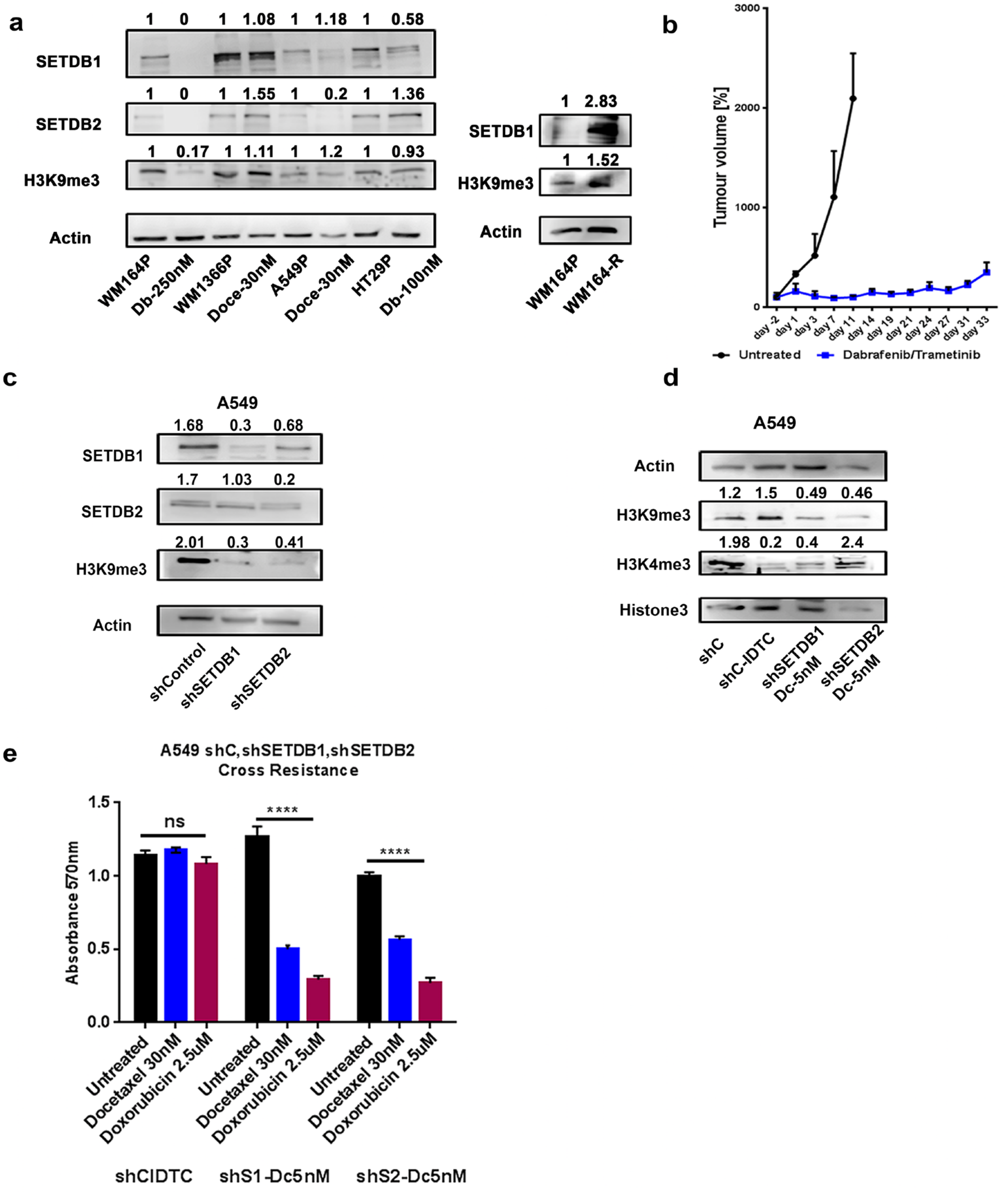

Supplementary Figure 8: Dynamics and functional studies of SETDB1 and SETDB2. **(a)** WM164, WM1366, A549, and HT29 cells were exposed to 200nM dabrafenib, 30nM docetaxel, 2.5µM doxorubicin, or 200nM dabrafenib respectively for 72 hours. Proteins were isolated and immunoblotted for SETDB1, SETDB2, and H3K9me3 (left). WM164 were treated with 50nM dabrafenib for 120 days and immunoblotted for SETDB1 and H3K9me3 (right). Actin was used as a loading control. All western blot images were quantified by ImageJ software. Values were normalized by subtracting from loading control. **(b)** C57BL6SCID mice were injected with $1x10^6$ WM164 melanoma cells subcutaneously. Mice were treated with dabrafanib (10mg/Kg) and trametinib (0.1mg/Kg) after formation of tumour (200mm^3). Tumour growth was monitored in each group (n=5) once every week for the entire period of the experiment. **(c)** A549 cells were transduced with non-target shRNA control and shRNA specific for SETDB1 and SETDB2. Protein lysates of the transduced cells were subjected to immunoblotting for SETDB1, SETDB2, Actin, and H3K9me3. **(d)** A549 shcontrol, shSETDB1, shSETDB2 transduced cells were exposed for 16 days to 5nM of docetaxel and probed for H3K4me3 and H3K9me3 by immunoblotting. **(e)** Same as in **(d)** but in addition challenged with toxic concentrations of docetaxel (30nM) and doxorubicin (2.5µM). Cell survival was analysed by MTT. Statistical analysis was performed by two-way ANOVA test and P-value is represented as (*) where, ****P≤0.0001.All western blot images were quantified by ImageJ software. Values were normalized by subtracting from loading control.

Supplementary Table 1: Titration of drug dosages for generating IDTCs

Cell line	Origin	Drugs	Concentration of Drugs	Sub-lethal concentration of IDTC
WM1366	Melanoma	Docetaxel	2.5, 5, 10nM	5nM
A549	Lung	Doxorubicin	500nM, 1uM, 2.5uM	500nM
		Docetaxel	2.5, 5, 10nM	5nM
HT29	Colon	Dabrafanib and Trametinib	15, 25, 40nM and 10nM Trametinib	25nM Dabrafenib and 10nM Trametinib
HCC827	Lung	Erlotinib	10, 20, 30nM	15nM
SKBR3	Breast	Docetaxel	2.5, 5, 7.5, 10nM	5nM
		Lapatanib	7.5,10,15, 20uM	Insensitive
WM164	Melanoma	Dabrafanib and Trametinib	15, 25, 40nM and 10nM Trametinib	25nM Dabrafenib and 10nM Trametinib

Different cancer cell lines were exposed to either chemo- or molecular therapy. At least three different concentrations were taken for each drug ranging from a lower to higher dosage that had the most growth inhibiting effect but still allowed cancer cells to survive as listed in Table 1. Every experiment was done in triplicate before selecting a sub-lethal dose for generating IDTCs for each of the cancer cell lines tested. A sub-lethal dose was defined that caused growth arrest but yet 70-80% cells survived after 12-15 days of drug treatment.

Supplementary Table 2: Differential expression of H3K9me3 modifiers in IDTCs

H3K9me3	WM1366	WM164	A549	HT29
SETDB1	NS	1.51	NS	NS
SETDB2	NS	2.6	2.52	NS
G9a	NS	NS	NS	NS
PRDM3	NS	NS	5.3	NS
KDM3A	-1.8	NS	NS	NS
KDM4A	NS	NS	NS	-1.57

H3K9 Methyl transferases- SETDB1, SETDB2, G9a, PRDM3
H3K9 Demethylases- KDM3A, KDM4B

Supplementary Table 3: RNA-seq expression data of the match-patient sample A-pre-treatment, B-early after treatment (14-16days)

Tags	Symbol	Entrez Gene Name	Location	Type(s)	10A	10B	12A	12B	13A	13B	16A	16B	19A	19B		
ENSG00000106462	EZH2	enhancer of zeste 2 polycomb repressive complex 2 subunit	Nucleus	transcription regulator	7.03	6.43	1.25	2.54	6.59	7.11	11.67	4.8	0.9	3.12		
ENSG00000143379	SETDB1	SET domain, bifurcated 1	Nucleus	enzyme	18.75	24.86	12.49	13.26	11.03	16.08	19.99	24.47	7.09	11.05		
ENSG00000136169	SETDB2	SET domain, bifurcated 2	Nucleus	enzyme	10.74	13.78	6.67	10.56	5.94	8.56	6.49	12.28	2.31	4.42		
ENSG00000117139	KDM5B	lysine (K)-specific demethylase 5B	Nucleus	transcription regulator	8.67	5.2	5.41	4.8	2.41	3.87	15.4	9.82	2.66	4.98		
**Tags**	**Symbol**	**Entrez Gene Name**	**Location**	**Type(s)**	**24A**	**24B**	**2A**	**2B**	**34A**	**34B**	**6A**	**6B**	**7A**	**7B**	**9A**	**9B**
ENSG00000106462	EZH2	enhancer of zeste 2 polycomb repressive complex 2 subunit	Nucleus	transcription regulator	18.54	8.3	5.86	2.71	3.34	5.18	16.53	4.18	9.4	2.59	15.73	1.42
ENSG00000143379	SETDB1	SET domain, bifurcated 1	Nucleus	enzyme	12.88	14.6	7.33	7.31	15.02	16.01	9.84	7.26	8.29	9	17.34	7.83
ENSG00000136169	SETDB2	SET domain, bifurcated 2	Nucleus	enzyme	8.69	15	6.48	4.36	3.49	5.57	3.37	4.14	2.88	7.33	4.93	4.75
ENSG00000117139	KDM5B	lysine (K)-specific demethylase 5B	Nucleus	transcription regulator	8.67	10.49	4.43	3.99	2.58	7.64	8	4.82	4.27	4.78	6.76	3.32

Supplementary method:

Reverse Phase Protein Array (RPPA): RPPA was performed as described earlier [2]. Briefly, Parent and IDTC samples were suspended in RPPA lysis buffer containing 1% Triton X-100, 50mM Hepes pH 7.4, 150mM NaCl, 1.5mM MgCl2, 1mM EGTA, 100mM NaF, 10mM Na Pyruvate, 1mM Na3VO4, 10% glycerol, and a cocktail of protease and phosphatase inhibitors. Samples were sonicated in an ice water bath for 20 minutes, and then were centrifuged at 14,000 RPM at 4°C for 20 minutes to isolate the protein supernatant. Protein samples were probed with 304 antibodies by tyramide-based signal amplification approach and visualized by DAB colorimetric reaction.

SUPPLEMENTARY REFERENCES

1. Menon DR, Das S, Krepler C, Vultur A, Rinner B, Schauer S, Kashofer K, Wagner K, Zhang G, Rad EB, Haass NK, Soyer HP, Gabrielli B, et al. A stress-induced early innate response causes multidrug tolerance in melanoma. Oncogene. 2015; 34:4448-59.
2. Hennessy BT, Lu Y, Gonzalez-Angulo AM, Carey MS, Myhre S, Ju Z, Davies MA, Liu W, Coombes K, Meric-Bernstam F, Bedrosian I, McGahren M, Agarwal R, et al. A Technical Assessment of the Utility of Reverse Phase Protein Arrays for the Study of the Functional Proteome in Non-microdissected Human Breast Cancers. Clin Proteomics. 2010; 6: 129-51. doi: 10.1007/s12014-010-9055-y.
